# BL-7010 Demonstrates Specific Binding to Gliadin and Reduces Gluten-Associated Pathology in a Chronic Mouse Model of Gliadin Sensitivity

**DOI:** 10.1371/journal.pone.0109972

**Published:** 2014-11-03

**Authors:** Justin L. McCarville, Yotam Nisemblat, Heather J. Galipeau, Jennifer Jury, Rinat Tabakman, Ad Cohen, Esmira Naftali, Bela Neiman, Efrat Halbfinger, Joseph A. Murray, Arivarasu N. Anbazhagan, Pradeep K. Dudeja, Alexander Varvak, Jean-Christophe Leroux, Elena F. Verdu

**Affiliations:** 1 Farncombe Family Digestive Health Research Institute, McMaster University, Hamilton, Ontario, Canada; 2 BioLineRx, Ltd., Jerusalem, Israel; 3 Division of Gastroenterology and Hepatology, Mayo Clinic, Rochester, Minnesota, United States of America; 4 Division of Gastroenterology and Hepatology, Department of Medicine, University of Illinois and Chicago and Jesse Brown VA Medical Center, Chicago, Illinois, United States of America; 5 Chromatography Unit, Scientific Equipment Center, The Mina and Everard Goodman Faculty of Life Sciences, Bar-Ilan University, Ramat-Gan, Israel; 6 Institute of Pharmaceutical Sciences, Department of Chemistry and Applied Biosciences, ETH Zürich, Zürich, Switzerland; CNR, Italy

## Abstract

Celiac disease (CD) is an autoimmune disorder in individuals that carry DQ2 or DQ8 MHC class II haplotypes, triggered by the ingestion of gluten. There is no current treatment other than a gluten-free diet (GFD). We have previously shown that the BL-7010 copolymer poly(hydroxyethyl methacrylate-*co*-styrene sulfonate) (P(HEMA-*co*-SS)) binds with higher efficiency to gliadin than to other proteins present in the small intestine, ameliorating gliadin-induced pathology in the HLA-HCD4/DQ8 model of gluten sensitivity. The aim of this study was to investigate the efficiency of two batches of BL-7010 to interact with gliadin, essential vitamins and digestive enzymes not previously tested, and to assess the ability of the copolymer to reduce gluten-associated pathology using the NOD-DQ8 mouse model, which exhibits more significant small intestinal damage when challenged with gluten than HCD4/DQ8 mice. In addition, the safety and systemic exposure of BL-7010 was evaluated *in vivo* (in rats) and *in vitro* (genetic toxicity studies). *In vitro* binding data showed that BL-7010 interacted with high affinity with gliadin and that BL-7010 had no interaction with the tested vitamins and digestive enzymes. BL-7010 was effective at preventing gluten-induced decreases in villus-to-crypt ratios, intraepithelial lymphocytosis and alterations in paracellular permeability and putative anion transporter-1 mRNA expression in the small intestine. In rats, BL-7010 was well-tolerated and safe following 14 days of daily repeated administration of 3000 mg/kg. BL-7010 did not exhibit any mutagenic effect in the genetic toxicity studies. Using complementary animal models and chronic gluten exposure the results demonstrate that administration of BL-7010 is effective and safe and that it is able to decrease pathology associated with gliadin sensitization warranting the progression to Phase I trials in humans.

## Introduction

Celiac disease (CD) is an autoimmune, chronic small intestinal enteropathy triggered by the consumption of gluten, composed of gliadins and glutenins, in genetically predisposed individuals expressing the HLA genes DQ2 or DQ8 [1], [2]. Development of the disease may be accompanied by an unknown environmental risk factor [3], [4]. Immune responses in these individuals mainly involve reaction with gliadin-derived peptides, such as the 33-mer [5], that have been modified by the native human enzyme, transglutaminase 2 (TG2), allowing greater binding affinity to HLA DQ2 and DQ8 haplotypes on gut immune cells [6]. About 1% of the population worldwide is affected by CD [7] and its prevalence is currently on the rise [8]. CD is characterized by a degree of small intestinal abnormalities including villus atrophy in the small intestine, an increase in intraepithelial lymphocytes (IELs) in villi tips and increased antibody titers to TG2 in serum [9], [10].

Currently there is no effective pharmacological treatment for CD. Individuals with CD must adhere to a strict regimen of gluten-free foods [11], [12]. Following a gluten-free diet (GFD) is difficult, and non-adherence can lead to ongoing symptoms, even when consuming as little as 50 mg/day [13], which is equivalent to a breadcrumb. Individuals with CD on a gluten containing diet have been shown to have nutritional deficiencies, neurological conditions, osteoporosis, increased mortality and higher risk for certain malignancies [7], [12], [14]. In addition, adult patients with CD that have adopted a GFD often experience residual symptoms or have evidence of persistent mucosal inflammation [15], [16]. Therefore there is need for supportive therapy to a GFD. There are a number of therapeutic approaches under development for CD. Some of these include intestinal permeability modulators and endopeptidases that cleave gliadin peptides making them less immunogenic [17]. Some therapies are based on blocking or skewing the immune response to gliadin peptides (eg. HLA-DQ2 blockers) and vaccination with gluten components [18].

Our therapeutic approach involves the use of a high molecular weight, non-absorbable polymer, BL-7010, previously called P(HEMA-*co*-SS), which binds gliadin intraluminally, protecting it from enzymatic cleavage by digestive enzymes, therefore avoiding immunogenic effects of gliadin-derived peptides. Our group has demonstrated that BL-7010 was effective at binding gliadin and decreasing gliadin/gluten-associated pathology *in vitro* using cell culture [19] and, in a pilot study *in vivo*, using the acute transgenic HLA-HCD4/DQ8 mouse model of gluten sensitization [19], [20]. We also provided preliminary evidence of activity upon prolonged exposure and, using tritium-labeled BL-7010 [20].

The purpose of the current study was to examine the effectiveness and safety of two batches of BL-7010, the previously described prototype polymer (hereby called Polymer A), that was found to be effective *in vitro* and *in vivo* in HCD4-DQ8 mice, and a new industrial batch (Polymer B), under chronic conditions of gluten sensitization. These two polymers have been produced under different synthesis methods. The reference polymer (Polymer B) was created by radical polymerization, whereas Polymer A was produced by an initiator and catalyst. These two polymers share a similar structure as used by Pinier *et al.* (2012) [20], and described by Liang *et al.* (2009) [21]. This comparison is key prior to the translation from preclinical to Phase I clinical trials using BL-7010. We chose to test this in a different model of gluten sensitivity, the NOD-DQ8 mouse that contains native mouse TCRs and develops moderate inflammation upon sensitization and chronic challenge with gliadin [22]. Additional aims were to determine the binding specificity and interactions of BL-7010 with gliadin and with nutrients that could be present in the small intestine, and to evaluate the genetic toxicity *in vitro* and the safety and systemic absorption of unlabeled BL-7010 as part of a 14-day repeated toxicology study in rats.

## Materials and Methods

### BL-7010 synthesis

BL-7010 co-polymer was synthesized by using two polymerization methods to form Polymer A and Polymer B of BL-7010. In the synthesis method of Polymer A, 2-hydroxyethyl methacrylate (HEMA) and sodium styrene sulfonate (SSNa) were polymerized in the presence of initiator and catalyst and then purified by filtration and precipitation to obtain the BL-7010 copolymer. The copolymer was obtained as white solid.

In the synthesis method of Polymer B, HEMA and SSNa were polymerized in the presence of a radical initiator. The work-up for the purification of the polymer was performed by few precipitation steps and finalized by dissolving the precipitation in water and drying. BL-7010 Polymer B was obtained as white solid with almost a double yield of Polymer A.

### Binding studies using surface plasmon resonance (SPR)

BL-7010 was synthesized by radical polymerization method. Binding of the BL-7010 to the Biacore chip was conducted via biotin-streptavidin interaction using biotinylated BL-7010. Biotin was attached to the BL-7010 chain *via* coupling reaction using N, N′-Diisopropylcarbobiimide (DIC) and 4-Dimethylaminopyridine (DMAP) as a coupling agent. The reaction was performed in DMSO under inert condition of argon for 48 h. The product was isolated by work-up with water, filtration, dialysis (cut-off 7,000 Da) and freeze-drying to obtain the biotinylated BL-7010 as a white solid. Streptavidin was immobilized to the chip (CM5, GE Healthcare) at 200 µg/mL in immobilization buffer (10 mM sodium acetate pH 5.0) to reach values of 6000RU using flow rate of 10 µL/min for 1.5 minutes. Subsequently, free-non-reacted active sites on the chip were blocked with 1 M ethanolamine hydrochloride pH 8.5 at 10 µL/min for 7 min [23]. The interaction of BL-7010 with vitamins and enzymes was evaluated with Polymer A while the interaction with gliadin, extracted and purified from whole wheat gliadin (Sigma), was assessed with both forms of BL-7010, Polymers A and B. The analysis was performed using the Biacore T100 instrument (GE). Studies were performed at 25°C, using HBS-EP (10 mM HEPES, pH 7.4, 150 mM NaCl, 3 mM EDTA, 0.05% Polysorbate 20) as running buffer and PBS as the dilution buffer. Regeneration of active surfaces was accomplished by passing a solution of 1 mM NaOH (20 µL/min, 30 sec).

Binding of BL-7010 to the chip was conducted by running a solution of 200 µg/mL at a flow rate of 5 µL/min, for 10 min on the same channel. BL-7010 was linked with a value of 500-1000RU. The kinetics and affinity of interactions with gliadin (10-640 ng/mL), pepsin (500 and 1000 nM), pancreatin (500 and 1000 nM), bovine serum albumin (BSA), and vitamins B1, B2, B3, B5, B6, B9, B12, D3, E, A, K1 and C (10 and 100 µM), all obtained from Sigma, were assessed. For the SPR analysis, evaluation was undertaken a kinetic evaluation of 1∶1 binding model of Biacore Evaluation Software v. 2.0.3, where all relevant QC parameters, such as residuals, goodness of fit, and curvature, were met.

### Animals

The McMaster University Animal Care Committee approved all animal experiments following the guidelines prepared by the Canadian Council on Animal Care. The efficacy experiments were conducted using a transgenic mouse strain expressing HLA-DQ8, deficient in MHC class II, on a NOD background (NOD-DQ8s) bred at McMaster University. Mice were kept on a low-fat (4.4%), gluten-free diet (Harlan Laboratories). Sensitization began between 6 to 12 weeks of age. For toxicity studies in rats, the care or use of animals were reviewed and approved by WuXi AppTec's Institutional Animal Care and Use Committee (IACUC).

### Sensitization and treatment protocol

Pepsin, trypsin digested gliadin (PT-gliadin), for sensitization, was prepared as described previously [20]. Briefly, gliadin (Sigma-Aldrich) was dissolved in endotoxin-free 0.02 N HCl and subsequently digested with 1 g of pepsin (Sigma-Aldrich) in a 37°C water bath for 2 h. Following the incubation, the pH was adjusted to 7.4 using endotoxin-free 2 M NaOH and trypsin (Sigma-Aldrich) was added and boiled for 30 min as described previously [24]. The PT-gliadin was then stored at −20°C until use. Gluten (Sigma-Aldrich), for challenge, was prepared by dissolving in 0.02 M acetic acid and stored at room temperature.

Mice were split into 4 groups (8 male and 8 female per group): a) non-sensitized negative control (negative), b) sensitized positive control (gluten challenge), c) sensitized group receiving Polymer B and d) sensitized reference group receiving Polymer A. Prior to sensitization, mice were injected twice, intraperitoneally (IP), with 250 µg of anti-CD25 antibody (Leinco Technologies), one week apart to deplete CD4^+^CD25^+^Foxp3^+^ cells. Sensitization began when mice were 6 to 12 weeks old. Sensitized mice received, by gavage, a mixture of PT-gliadin (500 µg) and cholera toxin (CT) (25 µg) (List Biological Laboratories Inc.) once a week for 3 weeks. Following sensitization, mice were administered 2 mg of gluten by gavage, 3 times a week for 4 weeks. Mice received a gluten challenge 24 h before sacrifice. Mice receiving either Polymers A or B were gavaged with gluten and the polymer at a weight ratio of 1∶3 (6 mg of BL-7010) during the challenge period. BL-7010 was administered as an oral gavage (dissolved in saline) 5 min prior to the gluten challenge. BL-7010 solution was given at a constant dose volume of 60 mg/ml. Sensitization and analysis were performed in blinded fashion. Non-sensitized mice received only CT during sensitization (no gliadin) and 0.02 M acetic acid for challenge (no gluten).

### Histological and immunohistological analysis of small intestinal tissue

Proximal sections of the small intestine were fixed in 10% formalin, embedded in paraffin and H&E stained. Sections were evaluated for evidence of inflammation and the measurement of villus-to-crypt (V/C) ratios. CD3^+^ staining was carried out as previously described [25]. Briefly, paraffin embedded sections of the small intestine were stained with a CD3^+^ (1∶2000, Dako) overnight, followed by an incubation with HRP and visualization via the substrate 3-amino-9-ethylcarbazole. CD3^+^ IELs were counted for every 20 epithelial cells and averaged over 5 villi tips. Histology (4x and 10x magnification) and immunohistochemistry (20x magnification) were viewed via light microscopy (Olympus). Images were analyzed in Image Pro Plus and the average V/C ratio was calculated for each group.

### Intestinal permeability

Intestinal permeability was measured as described previously [20]. Briefly, a 5-cm section from the jejunum was removed during sacrifice, cut along the mesenteric border, rinsed and mounted in an Ussing chamber (exposed surface area of 0.6 cm^2^). The section was bathed in oxygenated Krebs buffer containing 10 mM glucose (serosal side) or 10 mM mannitol (luminal side) at 37°C. In order to measure the net active transport across the epithelium, a short circuit current (Isc) was applied through the tissue under voltage clamp conditions. Conductance (mS/cm^2^) was recorded 15 min afterwards. Once equilibrium was achieved, ^51^Cr-EDTA (Perkin Elmer) was added to the luminal buffer and serosal buffer (500 µL) was collected every 30 min for 2 h and replaced with fresh buffer to maintain optimal volume. The ^51^Cr-EDTA was measured via a scintillation counter (Beckman Coulter LS6500 Multi-Purpose Scintillation Counter).

### RNA extraction and real-time PCR

To quantitate the PAT-1 mRNA, total RNA was extracted from jejunal tissues from all the four groups using RNeasy mini kit (Qiagen) according to the manufacturer's instruction. The quality and quantity of total RNA were determined by Beckman DU640 spectrophotometer. Extracted RNA was amplified by Brilliant SYBR Green qRT-PCR Master Mix kit (Agilent Technologies, Santa Clara; CA) utilizing gene specific primer for PAT-1 (sense primer, 5′-AAATGGAGCTGCAGAGGAGA-3′; antisense primer, 5′ GCTGGAGCAGAAGAGAATGG-3′) and GAPDH (sense primer, 5'-TGTGTCCGTCGTGGATCTGA-3'; antisense primer, 5'-CCTGCTTCACCACCTCTTGAT-3'). The relative mRNA levels of PAT-1 were expressed as % of control normalized to GAPDH used as internal control gene.

### 14-Day toxicity study

The potential toxicity, assessment of the reversibility, persistence, or delayed occurrence of toxic effects of BL-7010 was tested in Sprague-Dawley (SD) rats administered by oral gavage for 14 days followed by 14-day recovery period. In addition, the toxicokinetics (TK) of BL-7010 was determined in satellite animals. Eighty (N = 80, F&M) rats were randomly assigned to 4 groups to determine the toxicity of BL-7010 (Polymer B) when administered once daily for 14 days by oral gavage at doses of 1000, 2000 or 3000 mg/kg/day. The control group was administered vehicle (saline). Study animals were 10 or 15/group, and TK animals were 3 in the control group and 9/group in the treated groups. Five of the study rats/group in the control and high dose groups were allocated for a two-week recovery period. Parameters evaluated during the study included clinical observation, body weight, food consumption, ophthalmology examinations, clinical pathology (serum chemistry, hematology, coagulation, and urinalysis), gross pathology, organ weight histopathology and TK.

### Genetic toxicity studies

The potential mutagenic activity of BL-7010 was evaluated in accordance with the Organisation for Economic Co-operation and Development (OECD) 471 and 476 guidelines using bacterial and mammalian cells.

The bacterial reverse mutation assay was conducted to evaluate whether BL-7010 would induce reverse mutations at the histidine locus of the *Salmonella typhimurium* tester strains TA98, TA100, TA1535, and TA1537 or at the tryptophan locus of Escherichia coli tester strain WP2uvrA. The assay was conducted in the presence and absence of metabolic activation. Parallel testing was conducted with the negative control (vehicle alone) and positive controls. The mean number of revertants for the test article plates was compared to the mean number of revertants of the negative control plates for each of the five tester strains.

For the mammalian cells, the mouse lymphoma L5178Y/TK+/− cell line, heterozygous at the thymidine kinase (TK) locus, was used. This testing was conducted by exposing the cells to the test article dilutions and negative control (saline) and positive controls. The treatment was performed for 4 hours in the presence and absence of metabolic activation and 24 h in the absence of metabolic activation.

### Evaluation of systemic exposure and urine levels using LC-MS/MS methods

Systemic exposure and urine level of BL-7010 following single and repeated administration was evaluated as part of the 14 days toxicology study. Sixty (60, 30/sex) satellite rats were assigned to 1 control group (3/sex) and 3 test article treated groups (9/sex/group) for the assessment of TK. Animals in test article treated groups were administered with the solution of BL-7010 (Polymer B) in vehicle by oral gavage at 3000 mg/kg/day for 14 days. Rats in the control group were dosed for 14 days with the vehicle only (0.9% sodium chloride for injection (saline)). Blood samples were collected at predose (0), 0.5, 1, 2, 4, 6, 8, 12, 24 on Day 1 and Day 14 and additional 48, 72 and 96 h postdose on Day 14 from 3 animals/sex/time point/group via the jugular vein as an alternating sampling design from the test article treated animals and only collected at 1, 4 and 24 h postdose from the control group animals. Samples were collected into appropriately labeled tubes containing K_2_EDTA (Becton Dickinson). TK animals were placed in metabolic cages for urine collection up to 96 h post dosing. Urine samples were frozen at ≤−60°C until analysis.

Plasma fraction was separated, aliquoted and stored at −60°C pending analysis. For BL-7010 determination in plasma and urine, samples were analyzed by validated liquid chromatography - tandem mass spectrometry LC-MS/MS bioanalytical methods with Low Limit of Quantification of 5.00 µg/mL and 10.0 µg/mL for plasma and urine, respectively. Briefly, these methods utilize 30 µL aliquots of urine or 50 µL aliquots of plasma with dilution extraction for urine and protein digestion followed solid-phase extraction by Oasis HLB plates (30 µm, 10 mg) from Waters for plasma. Polyethylene glycol (PEG) was used as an internal standard. Waters Xbridge™, BEH300, 3.5 µm (2.1 mm I.D. x 50 mm) chromatographic column and isocratic elution of Mobile Phase A 0.1%NH4OH in 2 mM NH4Ac in water and Mobile Phase B 0.1%NH4OH in 20 mM NH4Ac in MeOH/AcN (v∶v, 1∶1) were used for separation. Analysis was done by negative ion Turbo Ion Spray in the multi-reaction monitoring mode (MRM). The LC-MS/MS technique employed very high declustering potentials at the orifice of the mass spectrometer before the mass filters. In this procedure, collision induced dissociation at the orifice resulted in a partial decomposition of BL-7010 into lower molecular weight fragments. These fragments were detectable in the range of the mass spectrometer. These lower molecular weight ions were then utilized as parent ions that were further dissociated by MS/MS into characteristic product ions for detection.

### Statistical analysis

Statistical analysis was completed via an ANOVA coupled with a Bonferroni post-hoc test.

## Results

### BL-7010 interacts specifically with gliadin

**Figure 1:**
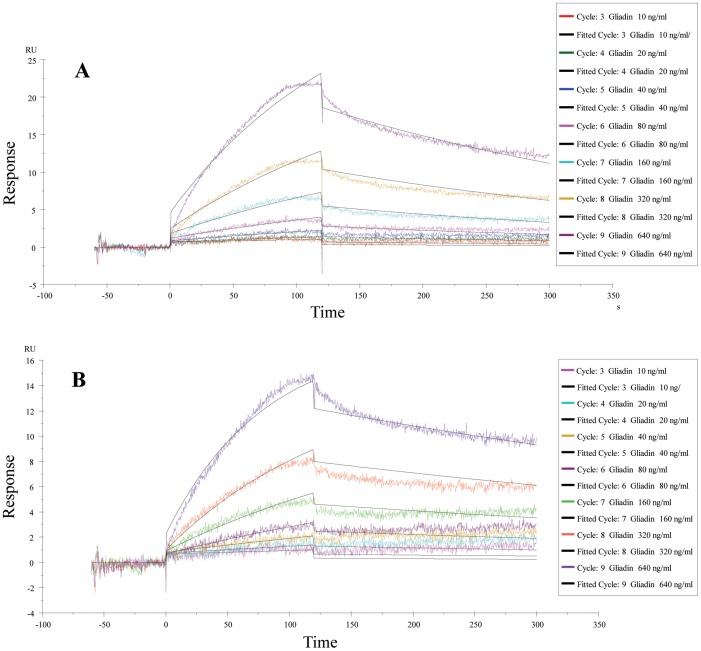
An overlay of sensograms showing Polymer A interaction with gliadin at a series of concentrations of 10-640 ng/mL. KD was determined to be 12.1 nM, with kon of 2.39×105 1/M·s and koff of 2.89×10-3 1/s. B. An overlay of sensograms showing Polymer B interacting with gliadin, with KD of 2.44 nM, kon of 6.11×105 1/M·s and koff of 1.49×10-3 1/s using Biacore Evaluation Software (see Methods). For both graphs, colored lines indicate experimental sensograms at various concentrations, while corresponding black lines denote fitting of experimental data using 1∶1 binding model with global Rmax.

We first evaluated the interaction of biotinylated BL-7010 Polymers A and B, with gliadin using the SPR technique. Polymers A and B showed high affinities for gliadin with KD values of 1.21 10^−8^ M (Chi^2^ of 0.263) (Figure 1A) and 2.44×10^−9^ M (Chi^2^ of 0.385) (Figure 1B), respectively. These results demonstrate that BL-7010 interacted avidly with gliadin. The differences in KD could be attributed to the slight composition and molecular weight differences between the two batches, which is related to the fact that polymers are characterized on an average basis and are not single molecular entities. It could be due to the different position of binding of the polymers to the chip since the biotin groups in the two polymers are positioned differently (one at the chain-end of the polymer and the other at numerous places on the main chain).

The interaction of biotinylated Polymer A with vitamins, BSA and digestive enzymes was evaluated by SPR. The enzymes pepsin and pancreatin were tested at two concentrations, 500 and 1000 nM, and gliadin was injected as a positive control at a concentration of 62.5 nM. No interaction between BL-7010 and pepsin or pancreatin, even at a 16-fold higher concentration than gliadin, was observed. Polymer A was found to interact with BSA with a KD of 1.09×10^−8^ M (Chi^2^ of 0.221). These results suggest that BL-7010 may also interact with human serum albumin but this is expected to have negligible effect on malnutrition and safety as discussed below. Due to their low molecular weights, the vitamins were evaluated at the high concentrations of 10 and 100 µM, while gliadin was injected as a positive control at a concentration of 100 nM. No binding of BL-7010 to the tested vitamins (K1, D, E, A, B5, C, B1, B2, B3, B6 and B12) was observed (Table 1).

**Table 1:** Interaction of BL-7010 (Polymer A) with gliadin, BSA, proteolytic enzymes and vitamins.

BL-7010 (Polymer B) – gliadin	**2.44E-9 M**
BL-7010 – gliadin	1.21E-8 M
BL-7010 – BSA	1.09E-8 M
BL-7010 – pepsin	No interaction^1^
BL-7010 – pancreatin	No interaction
BL-7010 – K1	No interaction
BL-7010 – D	No interaction
BL-7010 – E	No interaction
BL-7010 – A	No interaction
BL-7010 – C	No interaction
BL-7010 – B1	No interaction
BL-7010 – B2	No interaction
BL-7010 – B3	No interaction
BL-7010 – B5	No interaction
BL-7010 – B6	No interaction
BL-7010 – B12	No interaction

1 “No Interaction” means that no interaction signal (no increase in resonance units) was detected while increasing the concentration of the tested material and therefore no kinetics parameters could be calculated.

### BL-7010 attenuates gluten-induced pathology in a chronic mouse model

**Figure 2:**
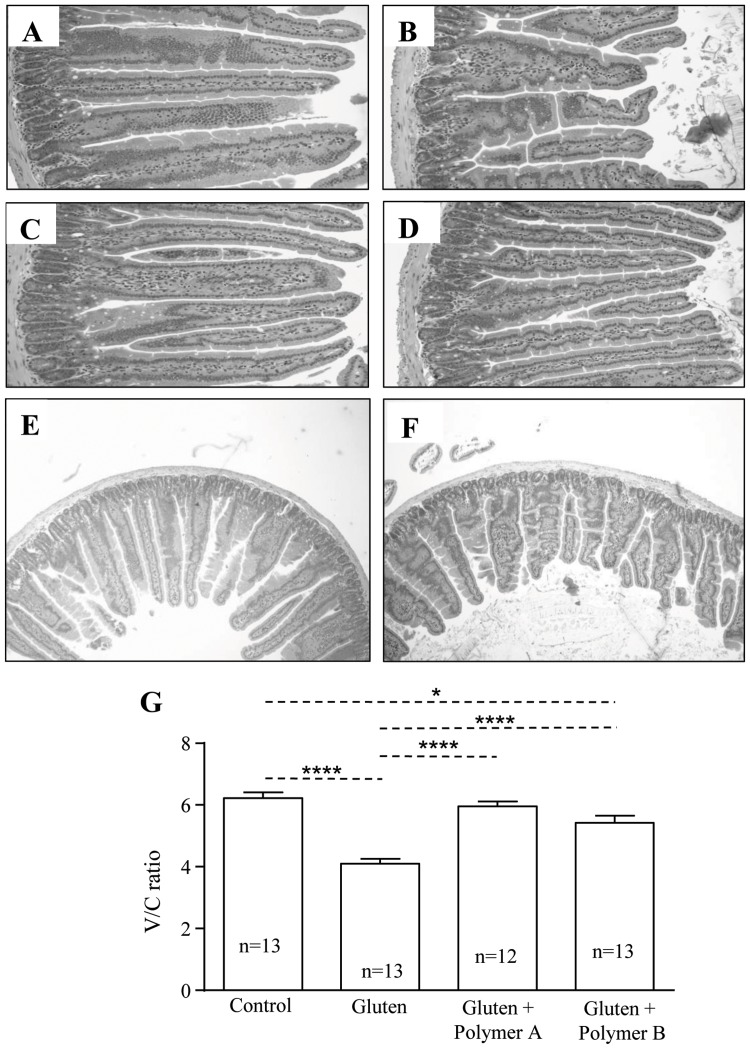
Administration of BL-7010 (Polymers A and B) at a gluten: BL-7010 ratio of 1∶3 decreased gluten-associated histological pathology in the small intestine of sensitized mice. Small intestinal H&E stained sections from (A) non-sensitized mice (control) (B) gliadin-sensitized mice (gluten) (C) gliadin-sensitized mice, receiving Polymer A (D) gliadin-sensitized mice, receiving Polymer B. (E) non-sensitized mice (control) at different magnification (F) sensitized mice at different magnification (G) Quantification of V/C ratios from small intestinal villi. H&E sections were viewed via optical microscopy (4x and 10x magnification). Bars represent the mean +/− SEM, statistical analysis was completed via one-way ANOVA and Bonferroni's post-hoc test (*p<0.05, ****p<0.0001) (n = 12–13).

Villus-to-crypt (V/C) ratios were decreased by 66% in sensitized mice without treatment in comparison to the non-sensitized (control) mice (p<0.0001) due to villus shortening (Figure 2). Administration of BL-7010 Polymer B prevented the reduction in V/C ratios induced by gluten challenge in comparison to sensitized mice that did not receive BL-7010. However, V/C ratios were still slightly lower than in non-sensitized mice (p<0.05). On the other hand, BL-7010 Polymer A prevented reductions in V/C ratio (p<0.0001) in comparison to gluten challenged mice and led to V/C ratios similar to those found in non-sensitized controls (Figure 2).

**Figure 3:**
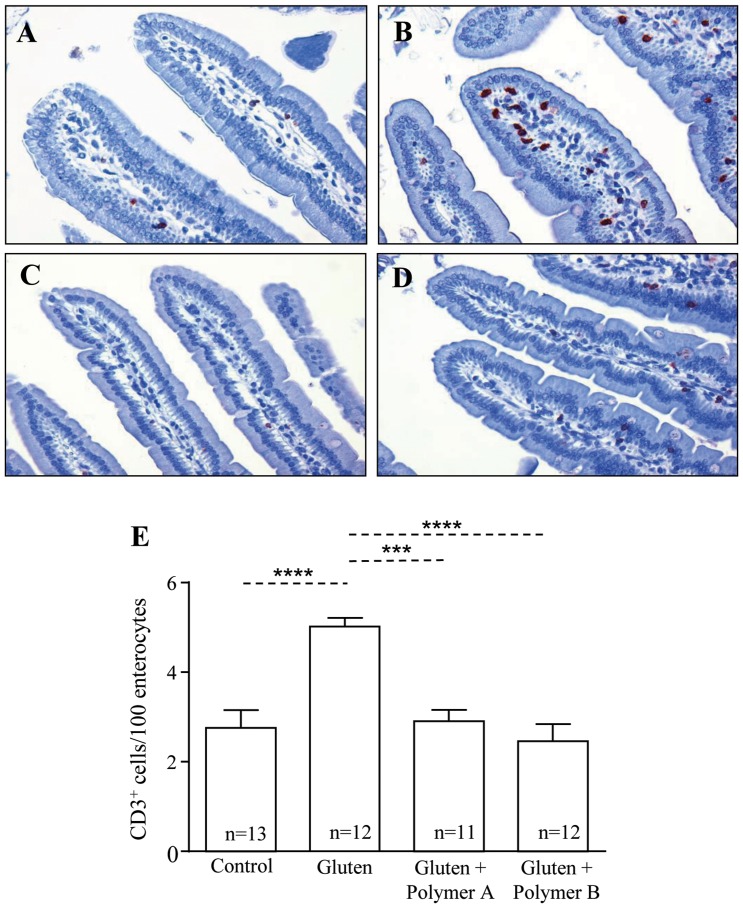
Administration of BL-7010 (Polymers A and B) decreased CD3^+^ intraepithelial cells in villi tips of gliadin-sensitized mice. Small intestinal CD3^+^ stained small intestinal tissues from (A) non-sensitized mice (control) (B) gliadin-sensitized mice (gluten) (C) gliadin-sensitized mice, receiving Polymer A (D) gliadin-sensitized mice, receiving Polymer B. (E) Quantification of CD3^+^ cells/100 enterocytes in villi tips. Stained sections were viewed via optical microscopy (20X magnification). Bars represent the mean +/− SEM, statistical analysis was completed via one-way ANOVA and Bonferroni's post-hoc test (***p<0.001, ****p<0.0001) (n = 11–13).

Gliadin-sensitized non-treated mice also had increased CD3^+^ IELs in villi tips of proximal small intestinal tissue in comparison to non-sensitized controls (p<0.0001) (Figure 3). Both Polymers B (p<0.0001) and A (p<0.001) were effective at lowering CD3^+^ IELs in comparison to gliadin-sensitized control mice. The results show that the activities of both batches of BL-7010 are comparable and confirm the ability of BL-7010 to attenuate pathological changes associated with CD.

### BL-7010 attenuates gluten-induced mucosal barrier dysfunction in a chronic mouse model

**Figure 4:**
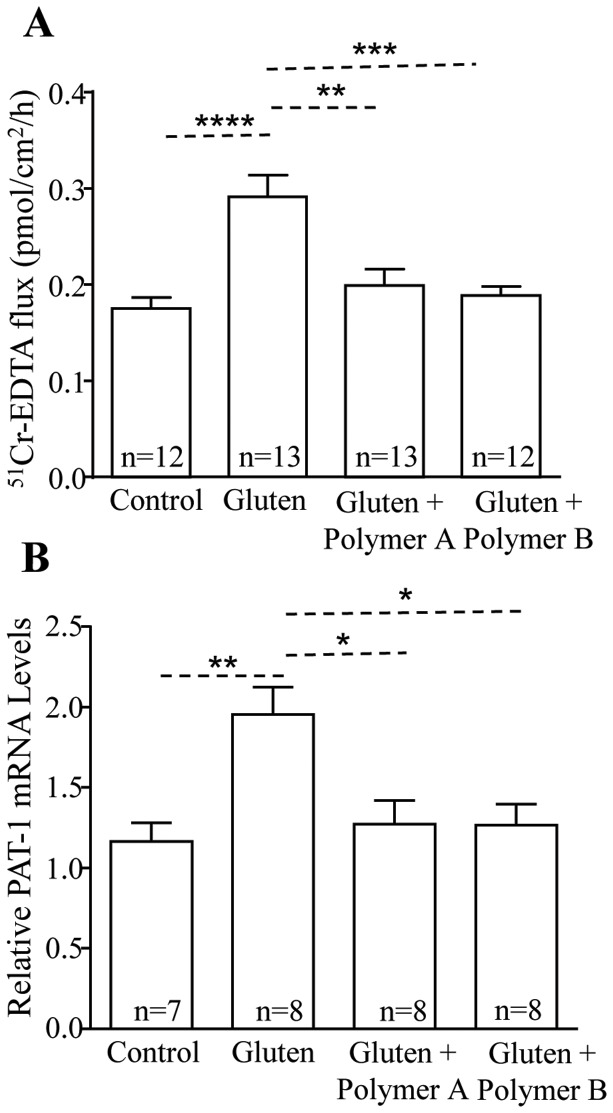
Administration of BL-7010 (Polymers A and B) decreased paracellular permeability and PAT-1 mRNA levels in gliadin-sensitized mice. (A) Gliadin-sensitized mice had higher ^51^Cr-EDTA flux in comparison to non-sensitized control mice, and administration of Polymers A or B decreased it to the same level as the non-sensitized control mice. (B) Gliadin-sensitized mice had higher mRNA levels of PAT-1 in comparison to non-sensitized control mice, and administration of Polymers A or B to sensitized mice decreased expression to the same level as non-sensitized control mice. Bars represent the mean +/− SEM, statistical analysis was completed via one-way ANOVA and Bonferroni's post-hoc test (**p<0.01, ***p<0.001, ****p<0.0001) (n = 12–13).

Sensitized non-treated mice had higher paracellular permeability in comparison to the non-sensitized mice (p<0.0001) (Figure 4A). Sensitized mice treated with Polymers A (p<0.01) or B (p<0.001) had lower paracellular permeability compared to the non-treated sensitized mice. BL-7010 treated mice had ^51^Cr-EDTA flux levels that were comparable to non-sensitized control mice. Gliadin-sensitized non-treated mice also exhibited an increase in PAT-1 mRNA expression in comparison to non-sensitized control mice (p<0.001) (Figure 4B). Gliadin-sensitized mice receiving Polymer A or Polymer B had lower PAT-1 mRNA expression in comparison to sensitized non-treated mice (p<0.05), and comparable to that of the non-sensitized mice.

### BL-7010 is not absorbed systemically

The presence of BL-7010 in rat plasma and urine was evaluated post single and 14-days once daily repeated administration of Polymer B at a dose of 3000 mg/kg/day. This dose is ∼23 times higher than the expected effective human dose. BL-7010 was not found in any plasma sample tested following single or 14 days repeated administration of 3000 mg/kg, suggesting the lack of systemic exposure. BL-7010 was found at very low levels in the urine (0–<1.54% of dose) (data not shown). The trace amounts found in the urine are due to external contamination with feces in the metabolic cages.

### BL-7010 safety and tolerability

Oral administration of BL-7010 once daily for 14 consecutive days was well tolerated at all dose levels. No mice reached endpoint to be sacrificed due to adverse effects. Treatment with BL-7010 at doses up to and including 3000 mg/kg/day did not cause adverse effects on body weight, food consumption, clinical pathology, ophthalmoscopy, organ weights, or gross pathology following the dosing or recovery phases. BL-7010 was clinically well tolerated at 1000, 2000, and 3000 mg/kg/day when administered by oral gavage to rats for 14 days. The no observed adverse effect level (NOAEL) was considered to be 3000 mg/kg/day for both males and females. In the bacterial reverse mutation assay BL-7010 was considered to be nonmutagenic to *S. typhimurium* tester strains TA98, TA100, TA1535, and TA1537, and to *E. coli* tester strain WP2uvrA. In the mammalian cells mouse lymphoma assay the test article did not cause a two-fold or greater increase in the mean mutant frequency over the negative control of the L5178Y/TK+/− cell line either in the presence or absence of metabolic activation and therefore is not consider mutagenic in this assay.

## Discussion

There are several approaches for developing treatments for CD. One of them is the degradation of immunogenic gluten peptides to non-immunogenic fragments in the GI tract. This approach was found to be clinically effective [26] in patients receiving gluten degrading proteases proving that prevention of the formation of gliadin immunogenic peptides is clinically beneficial. In contrast to the enzymatic degradation approach, BL-7010 prevents the formation of gliadin immunogenic peptides by sequestering gliadins in the GI tract [19], [20].

The main objectives of this paper were to evaluate the efficacy of BL-7010, in NOD-DQ8 mice, a mouse model that develops moderate inflammation in the small intestine when sensitized and challenged with gluten [22]. We also tested the activity of 2 batches of polymer. Compared to classical low molecular weight drugs, polymers are not characterized by a precise molecular structure but are defined on an average fashion with respect to their composition and size. It is therefore important to validate the reproducibility of the scaled up manufacturing process suitable prior to initiating clinical trials in humans. In addition, the previous *in vivo* study [16] demonstrated the efficacy of Polymer A, which was synthesized by a preliminary synthesis method. The yield and cost effectiveness of this process was relatively poor. Therefore, we have developed a new synthesis method (Polymer B) which has improved cost effectiveness. Due to the fact that Polymer B has slightly different characteristics than Polymer A, the product of the preliminary synthesis method, we aimed to confirm its activity in an animal model of gluten sensitivity. Polymer A was used as a control in the *in vivo* and *in vitro* binding studies, as it was proven to be effective in the past [21]. In the current study we showed that the two batches of BL-7010, Polymers A and B, were effective at abrogating gluten-associated pathology in gliadin-sensitized NOD-DQ8 mice. Both batches of BL-7010 prevented the decrease in V/C ratio, decreased intraepithelial lymphocytosis and intestinal epithelial function in small intestinal tissues.

Recruitment of IELs and decreased V/C ratio, represent pathological features of CD [11], [27]. Both batches of BL-7010 prevented changes in IELs and V/C ratios induced by gliadin, indicating amelioration of morphological changes as well as prevention of innate immune parameters in this model. Individuals with CD have been shown to have intestinal barrier dysfunction, which emerges early in the disease [28]. Our results demonstrated that BL-7010 prevented barrier dysfunction associated with gliadin sensitization. A leaky barrier may contribute to uptake of gliadin [29] or other proinflammatory luminal antigens of microbial origin [30], and promote progression of disease. Thus, drug development has targeted modulation of the intestinal barrier in CD, particularly using larazotide acetate [31]. This drug has demonstrated the ability to block some symptoms due to gluten sensitivity [31] via actin rearrangement and has also demonstrated the ability to decrease *in vitro* [32] and *in vivo* [33] permeability. BL-7010 normalized paracellular permeability most likely through an indirect mechanism, by reducing production of toxic peptides, limiting interaction of these peptides with the gut mucosa and through prevention of induction of immune responses. In addition to functional analysis of the intestinal epithelium, we measured ion transporter mRNA expression. PAT-1 mRNA expression, a small intestinal luminal membrane CL-/HCO_3_- exchanger [34] primarily localized to the villi of the small intestine [35], [36]. The expression of PAT-1 was increased in gliadin-sensitized mice and its high expression was restored to control levels in mice treated with both batches of BL-7010. The results suggest that ion transport mechanisms that are important for absorption and homeostatic mechanisms are affected by moderate gluten-induced inflammation and are normalized by BL-7010 administration, probably indirectly through prevention of the interaction of gliadin with the intestinal mucosa.

The mechanism of action of BL-7010 relies on electrostatic and hydrophobic interactions with gliadin and possibly on the formation of hydrogen bonds [21]. The relative specificity of BL-7010 to gliadin is a function of the amino acid sequence of gliadin and its resulting electrostatic charge and hydrophobicity. This kind of mechanism raises concerns about the specificity of BL-7010 to gliadin and possible side effects, such as malnutrition, which can result from interaction with nutrients, or an effect on the digestion process by interaction with digestive enzymes. Additionally, non-specific binding may reduce efficacy due to lower fraction of free BL-7010 that is available for neutralization of gliadin. However, the binding of BL-7010 to other wheat and barley fractions is of interest. We have previously shown that P(HEMA-*co*-SS) blocks the digestion of wheat gluten and barley hordein *in vivo* [20]. Incubation of P(HEMA-*co*-SS) with whole wheat gluten reduces the glutenin sequence SQQQQPPV by 60%, providing indirect evidence that BL-7010 may bind to glutenin. In this study, we demonstrated by SPR technique, that BL-7010 does not interact with the tested vitamins. No interaction was observed with the tested digestive enzymes, pepsin and pancreatin, implying that BL-7010 will not affect the digestion process. On the other hand, high affinity of BL-7010 to gliadin, for both polymeric batches, was observed. Some interaction of BL-7010 with BSA was noted but the fact that BL-7010 is not absorbed into the blood circulation and therefore is not expected to interact with human serum albumin, mitigate the risk of safety issues that can result from such interaction. The determination of KD constant by SPR to monitor the interactions between polymers and proteins should, however, be interpreted with caution since in solution the binder is not bound to a surface and therefore less conformationally hindered to interact with the protein. Our former data obtained by gel electrophoresis indicated that at pH 6.8, albumin interacted less than α-gliadin with BL-7010 [19]. A significant interaction of BL-7010 with nutrients seems therefore unlikely. In agreement with our previous study [30], we show here that a 14-day administration of a much higher dose was safe with no effects on body weight, food consumption, clinical pathology, ophthalmoscopy, organ weights, and gross pathology. This will nevertheless require confirmation in safety studies in humans.

Lack of systemic exposure is a key feature of BL-7010 that contributes to the safety and efficacy of this product. Due to its high molecular weight, BL-7010 is not anticipated to be absorbed systemically and it was found, using tritium-labeled BL-7010, that this compound is excreted almost completely in the feces, both in normal rats and in animals with intestinal barrier dysfunction, gliadin-challenged HLA-HCD4/DQ8 mice [20]. In the tritium-labeled study some radioactivity was detected in the urine (less than 2%), most likely due to proton exchange with water. Thus, in this study, we established a sensitive LC/MS/MS method that can specifically detect BL-7010 in plasma and urine at very high sensitivity and does not rely on radiolabeling, therefore excluding possible bias caused by proton exchange. We showed that BL-7010 is not found in the rat plasma of animals receiving single or 14 days repeated treatment at extremely high dose levels. The low levels of BL-7010 found in the urine could be explained by contamination of the urine samples with feces in the metabolic cage. Indeed, BL-7010 was most commonly found in the urine after more than 8 h, correlating to the time it was found in the feces in previous studies [20], and in accordance with the animals' circadian cycle, activity time and eating period. As there is no simple method to completely separate feces and urine samples from animals, the question of systemic exposure should be further evaluated in clinical studies.

The safety of BL-7010 (Polymer B) was evaluated in a repeated GLP toxicology study. Repeated administration of up to and including 3000 mg/kg/day doses was well tolerated and safe. Microscopic evaluation of the oral cavity, tongue, larynx, esophagus, stomach, small intestine (duodenum, jejunum, ileum) and large intestine (cecum, colon, rectum) revealed no evidence of irritation in these tissues. The no observed adverse effect level (NOAEL) was considered to be 3000 mg/kg/day for both males and females. *In vitro* genetic toxicity studies showed that BL-7010 has no mutagenic activity in both assays, indicate that BL-7010 is not mutagenic. Polymer A was also evaluated in a 14-day repeated toxicity study in rats up to dose of 2000 mg/kg/day and was found to be completely safe (data not shown).

In summary, this study demonstrated that BL-7010 is effective at abrogating gluten-associated pathology in a model of chronic gliadin-sensitization using NOD-DQ8 mice. Both batches of BL-7010 were effective in this model, suggesting that the synthesis method of BL-7010 is robust and produce a functional polymer. Furthermore, the results indicate that BL-7010 may be safe for long term use, as systemic absorption and interactions with vitamins and digestive enzymes were absent. BL-7010 safety was confirmed at doses that are far higher than the expected clinical effective dose. BL-7010 is currently undergoing clinical trials to further evaluate its efficacy, safety and systemic exposure in celiac disease patients.

## Supporting Information

File S1 **Supplementary information.** Includes data for Figures 1, 2, 3, and 4.
(XLSX)
